# Unusual cause of congenital hypothyroidism in a term infant

**DOI:** 10.1136/bcr-2020-237930

**Published:** 2021-02-19

**Authors:** Hester Vlaardingerbroek

**Affiliations:** 1 Department of Pediatrics, Subdivision of Endocrinology, Willem Alexander Children's Hospital, LUMC, Leiden, The Netherlands; 2 Department of Pediatrics, Subdivision of Endocrinology, Sophia Children's Hospital, Erasmus Medical Centre, Rotterdam, The Netherlands

**Keywords:** thyroid disease, diet

## Abstract

Both insufficient and excessive maternal iodine consumption can result in congenital hypothyroidism. In East Asian cultures, seaweed is traditionally consumed in high quantities by peripartum women as it is thought to improve lactation. We present a case of transient congenital hypothyroidism due to maternal seaweed consumption at a daily basis during pregnancy and lactation in a Dutch family without Asian background. This case highlights that even in families of non-Asian background, high maternal intake of iodine-rich seaweed occurs and can result in transient or permanent hyperthyrotropinemia in the neonate with risk of impaired neurodevelopmental outcome if untreated.

## Background

Congenital hypothyroidism (CH) is a condition in which neonates have thyroid hormone deficiency. CH can be transient or permanent, depending on the aetiology and duration of hypothyroidism. Thyroidal causes such as agenesis or underdevelopment of the thyroidal gland (thyroid dysgenesis) or insufficient hormone synthesis (thyroid dyshormonogenesis) are the most common causes. Central CH due to absent or insufficient thyroid-stimulating hormone (TSH) secretion in the pituitary gland is less common. Other causes are TSH receptor insensitivity and transient CH. Without early detection and treatment, neurodevelopment and metabolism are severely hampered. We present a case with CH due to an unusual cause in non-Asian infants, illustrating the importance of a thorough patient history.

## Case presentation

**Figure 1:**
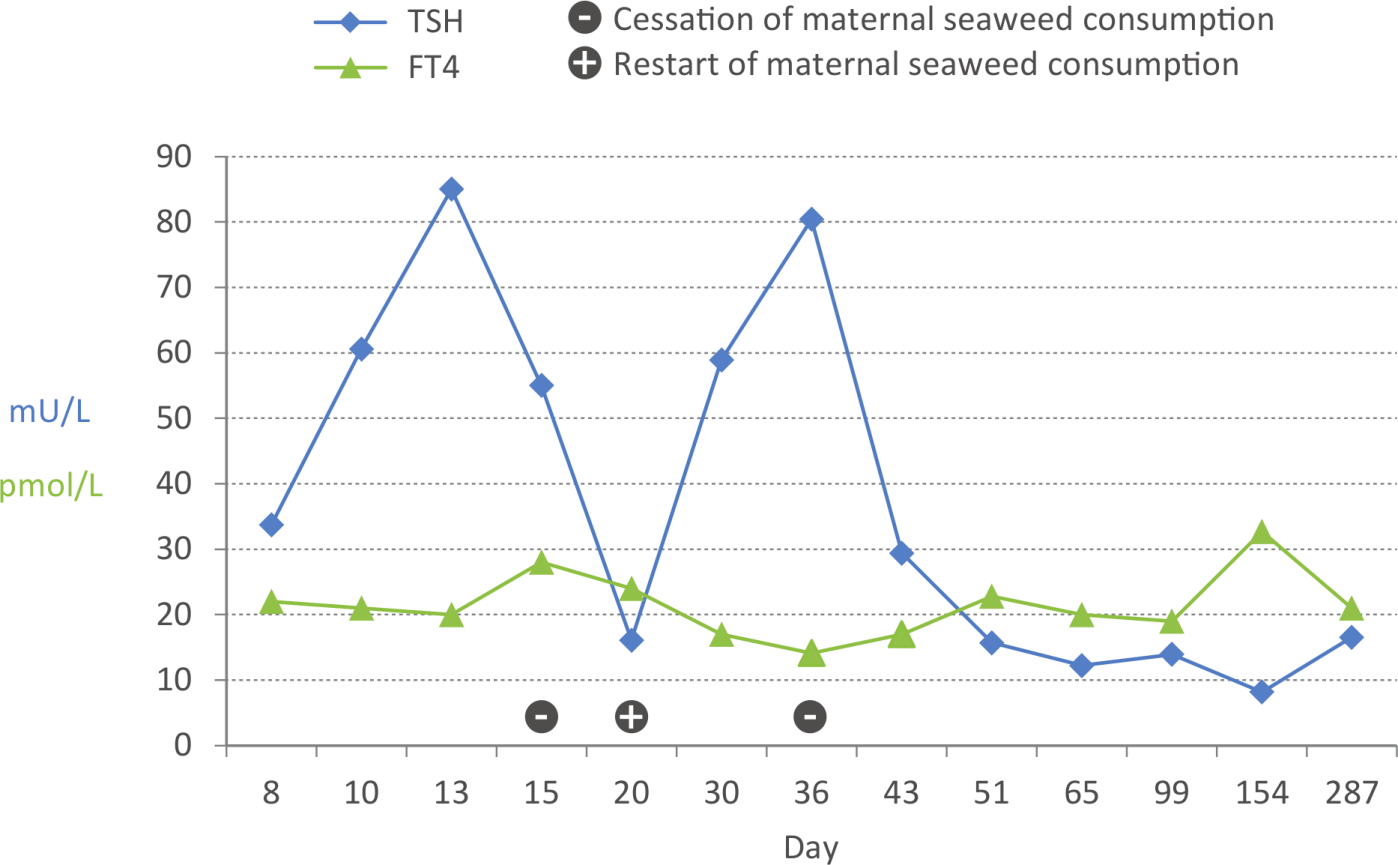
Trend in FT4 and TSH from birth onwards, illustrating the effect of maternal seaweed consumption on TSH concentration. FT4, free T4; TSH, thyroid-stimulating hormone.

We present a Dutch Caucasian boy who was referred at day 8 of life for abnormal CH screening with a T4 of 72 nmol/L blood (~144 nmol/L serum) with a SD of −0.9 on average in the batch of measurements made that day (reference, >−1.6 SD) and a TSH of 23 mE/L (reference, <7 mE/L). Pregnancy and delivery were uneventful; he was born at 38 weeks and 5 days of gestation with a body weight of 3350 g. The family history revealed no thyroidal problems, and he has a healthy brother. The boy is a peaceful baby and drinks breast milk ad libitum. His body weight decreased until day 6 and started to increase thereafter. Temperature and defecation were unremarkable. Hearing screening was initially insufficient, but normal at a repeat measurement. Except for mild jaundice that did not require phototherapy, physical examination revealed no abnormalities: especially, his pulse rate was normal and thyroidal enlargement was not palpable. Laboratory results showed repeatedly increased TSH concentrations, with free T4 (FT4) in the normal range, also described as hyperthyrotropinemia (figure 1). Our differential diagnosis was mild dyshormogenesis, partial TSH receptor insensitivity or transient causes of CH. At day 15, we performed a ^123^I-perchlorate discharge test, which displayed normal ^123^I uptake; however, after perchlorate administration, the uptake increased instead of decreased, suggesting a highly stimulated thyroid gland despite FT4 in the high-normal range. Ultrasound of the thyroidal gland performed at day 51 showed a normally sized and located thyroid gland without focal abnormalities.

At this point, history was repeated, which revealed that the mother consumed seaweed at a daily basis throughout pregnancy and lactation. She consumed a bowl of wakame miso soup daily, hijiki or arame seaweed once a week and nori seaweed once a week. In addition, she used chlorella (algae powder) and ‘sweet iron’ supplements during pregnancy. Her motivation for daily seaweed consumption was the perceived health aspect.

Due to the lack of adequate reference ranges for mother’s milk and the lack of upper levels in urine that can cause hyperthyrotropinemia in the fetus and infant, we decided not to measure iodine concentration in mother’s milk or urine. To exclude other causes of CH, we performed mutation analysis of the THRB gene and a gene panel for thyroidal CH, including the sequencing and copy number variance analysis of the following genes: CDCA8, DUOX1, DUOX2, DUOXA2, FOXE1, GLIS3, GNAS, HOXA3, IYD, JAG1, NKX2-1, PAX8, SLC26A4, SLC5A5, TG, TPO, TPST2 and TSHR. In these genes, no mutations or variations were found.

## Outcome and follow-up

Cessation of maternal seaweed consumption was strongly advised. After cessation, TSH concentrations gradually decreased but did not normalise. The FT4 concentrations remained in the normal range. As a result of TSH values being >10 mU/L, the mother was strongly recommended to supplement her infant with levothyroxine to prevent long-term complications. However, so far the mother was reluctant to start supplementation because of normal FT4 concentrations. During follow-up until 9 months of age, his neurodevelopment was normal. We will continue to follow up the thyroid function and development of this boy.

## Discussion

This case illustrates the importance of a detailed history including maternal iodine exposure, even in populations without cultural or ethnic background known for high seaweed consumption.

In our case, the excess maternal iodine during pregnancy and lactation due to daily seaweed consumption resulted in congenital transient hypothyroidism.

Direct iodine overload to the neonate can be caused by iodine-containing disinfection agents or contrast medium administration during the perinatal period or can be caused by iodine-rich medication or diet. Maternal exposure to iodine can result in high iodine concentrations in the fetus and newborn due to placental transfer and/or by secretion of iodine in breast milk.^1 2^ The thyroid possesses protective mechanisms for excess iodine: the so-called Wolff-Chaikoff effect. In cases of excess iodine, iodine uptake in the thyroid gland is blocked by a decreased activity of the iodine symporter, leading to reduced T4 and increased TSH concentrations. In adults, this blockage lasts on average for 48 hours.^3^ The fetus and neonate are very sensitive to excess iodine because the Wolff-Chaikoff effect is not functional until gestational week 36. The fetus membranes are highly permeable, and the fetus can absorb iodine from the amniotic fluid through the skin or gastrointestinal tract, or iodine is transferred through the placenta. In addition, iodine trapping processes in the thyroid gland are active, and iodine renal clearance is low.^4^ The duration of the blockage of iodine transport into the thyroid gland in neonates can last for weeks to months.^4^

In many East Asian cultures, seaweed soup is considered a healthy staple. Korean mothers are traditionally given large amounts of brown seaweed soup postpartum, up to 4–5 times a day in the first few days after delivery, to promote breast milk supply. The iodine content of seaweed products is highly variable. Especially seaweed types such as kelp, kombu and Sargassum have very high iodine contents and can contain >1000 µg of iodine per gram product.^5 6^ Harvesting, storage and cooking methods can greatly affect iodine content. It was reported that 250 mL of seaweed soup can provide 500–1700 µg of iodine, based on whether the seaweed itself is consumed or not.^7^

The Dutch recommended daily allowance (RDA) for iodine is based on Scandinavian RDAs (2012).^8^ For pregnant women, the RDA is considered to be 175 µg/day and during lactation to be 200 µg/day. No RDA for infants <6 months was established. An RDA for infants between 80 µg and 110 µg has been proposed by others.^9 10^ The European Food Safety Authority established the tolerable upper limit that is considered safe for adults to be 600 µg/day, and the maximal amount for infants aged <1 year is not established.^11^ The WHO proposed an upper limit for children aged <2 years to be 180 µg/day.^12^ Daily ingestion of iodine-rich seaweed products easily exceeds the daily recommended intake of iodine as in our case. In addition to iodine, seaweed contains several minerals, vitamins, soluble fibres and flavonoids which are considered to have beneficial effects on lifestyle-related diseases, but also contains heavy metals such as arsenic, which can have detrimental health effects.^13^

Published cases of CH due to excess maternal dietary iodine are limited to families with Asian background. In a Korean study, subclinical hypothyroidism in preterm infants was associated with high iodine concentrations in breast milk. These Korean postpartum women traditionally consumed brown seaweed soups, resulting in excess iodine in a third of infants.^14^ In a study in Japan in 34 neonates with abnormal CH screening, 44% were diagnosed with hyperthyrotropinemia, that is, high TSH, caused by excess maternal ingestion of iodine during pregnancy, of which 80% required levothyroxine because of hypothyroxinemia or persistent hyperthyrotropinemia. In addition, it was suggested that hyperthyrotropinemia due to excessive maternal iodide ingestion during pregnancy may be permanent.^4^ Possible risk factors for permanent hyperthyrotropinemia in these infants may be repeated excess iodine due to placental transfer, breast milk and baby food. In these infants, thyroid function may be permanently altered, possibly by an intrauterine iodine imprinting effect.^4^

In Australia, New Zealand, UK and USA, four cases of iodine-induced neonatal hypothyroidism due to maternal seaweed consumption are reported. In all of these four cases, the mothers had an Asian background.^15–17^ All these infants needed treatment with levothyroxine for a duration of 4 months to 2 years.

Whether or not to treat children with (permanent) hyperthyrotropinemia and normal FT4 concentrations is still a controversial subject. Levothyroxine therapy might be considered with persistent TSH concentrations above 10 U/L, mainly because of the potential long-term increased risk of cardiovascular morbidity and mortality. This is mainly investigated in Hashimoto-related hyperthyrotropinemia or other autoimmune diseases.^18^ In mothers with Graves’ disease, neonatal hyperthyrotropinemia is often transient.^19^ After maternal iodine consumption, neonatal thyroid function may be permanently altered, possibly by an intrauterine iodine imprinting effect, resulting in persistent hyperthyrotropinemia (with or without low FT4 concentrations) with potential negative effects on cardiovascular health.

Since TSH was spontaneously decreasing in our case, we decided not to supplement with levothyroxine yet.

This case illustrates the importance of a detailed history including maternal iodine exposure even in populations without cultural/ethnic background known for high seaweed consumption. Maternal seaweed consumption can result in transient or permanent hyperthyrotropinemia in the neonate with risk of neurodevelopmental outcome if untreated.

**Learning points**

- Maternal diet should be included in the history of all neonates referred for screening of abnormal congenital hypothyroidism.
- Even in families of non-Asian background, high maternal intake of iodine-rich seaweed occurs and can result in transient or permanent hyperthyrotropinemia in the neonate with risk of neurodevelopmental outcome if untreated.
- Food safety institutes, general healthcare providers, gynaecologists and midwives should advise pregnant and lactating women not to ingest iodine-rich foods on a daily basis because seaweed iodine content is highly variable and easily exceeds recommended daily allowances.
